# Maternal Mental Health During and Before the COVID-19 Pandemic: A Comparative Analysis in the Kingdom of Bahrain

**DOI:** 10.7759/cureus.46938

**Published:** 2023-10-13

**Authors:** Bessy Varghese, Amala Sunder, Waad Aldoseri, Taqwa Alsheglawi, Yusra Mirghani Aljailani Fadhulalla, Rawah Hashim Albadawi, Indira Kumar Natarajan, Abida Qureshi, Basma Darwish

**Affiliations:** 1 Obstetrics and Gynaecology, Bahrain Defense Force Hospital, West Riffa, BHR; 2 Obstetrics, Royal College of Surgeons in Ireland - Bahrain, Riffa, BHR; 3 Obstetrics, Bahrain Defense Force Hospital, West Riffa, BHR; 4 Stroke, National Health Services, Stanford, GBR; 5 Obstetrics and Gynaecology, Bahrain Defense Force Royal Medical Services, West Riffa, BHR

**Keywords:** mental health, depression, anxiety, covid-19 pandemic, hospital anxiety and depression scale (hads)

## Abstract

Introduction

The coronavirus disease 2019 (COVID-19) pandemic created a crisis in health care systems worldwide. The maternity services were restricted due to the pandemic regulations. The psychological burden on the pregnant women was to various extents. Individuals and organizations implemented support schemes to understand and support their mental health. In our study, the psychological impact of pregnant women who contracted COVID-19 during pregnancy was compared with pregnancy of the same population before the pandemic as it could be a précised and helpful method to counsel pregnant women effectually.

Study design

This retrospective study included 111 women and was conducted at Bahrain Defense Force Hospital from January 2021 until December 2021. The researchers distributed a Hospital Anxiety and Depression Scale (HADS) questionnaire to women who delivered babies during the pandemic. The researchers then analyzed these scores and compared them with the scores of a control group of women who completed their pregnancies before the pandemic. The data were analyzed using SPSS Version 25.0 (IBM Corp., Armonk, NY). P-values of less than 0.05 were considered statistically significant.

Results

The HADS questionnaire results demonstrated that women’s anxiety and depression during their pregnancy during the COVID-19 pandemic were significantly higher than that during their pregnancy before the pandemic, with a mean score of 14.97 (95% CI: 14.5 to 15.4) and 9.4 (95% CI: 8.8 to 9.9), respectively, and a *p*-value of <0.001. Additionally, during the COVID-19 pandemic, 100% of participants were “abnormal” in the anxiety category, and 86.5% were “abnormal” in the depression category, whereas before the pandemic, 0.9% of the studied population were abnormal, 3.6% were borderline abnormal, and 95.5% were normal in the depression category. The comparison of these scores highlighted that the pandemic had a significant negative psychological effect on the mothers during pregnancy, thus increasing their anxiety and depression. The correlated personal, social, and clinical factors were fear of delivery, fear of disease transmission, loss of family support, social isolation, uncertainty of life, and economic crises. Depression scores were significantly correlated to factors such as fear of disease transmission to the baby (p=0.027), fear of delivery (p=0.008), and loss of family support (p=0.001). Contributing factors and anxiety scores yielded significant correlations with fear of delivery (r_s _=0.258), fear of transmission (r_s_=0.198), and uncertainty of disease life (r_s_=0.247). As for depression, it was significantly correlated to one factor: loss of family support (r_s_=-0.335).

Conclusion

The mental health, in terms of anxiety and depression, of pregnant women was significantly affected during the COVID-19 pandemic.

## Introduction

The coronavirus disease 2019 (COVID-19) pandemic significantly impacted the mental health of women who were pregnant during this period. It is important to address issues of mental health in pregnant women not only to support their adaptation to physiological changes in their body but also for the well-being of the growing fetus. Anxiety and depression during the prenatal period may be related to adverse outcomes such as miscarriage, low birth weight, preterm birth, and low Apgar scores [1]. Additionally, prenatal stress is associated with cognitive, emotional, and behavioral problems in children after they are born [2]. The COVID-19 pandemic heightened fear and stress among pregnant women, culminating in psychological effects such as anxiety and depression. Studies demonstrate that contributing factors include disease spread, quarantine, inconvenience of antenatal checkups, restricted family support, economic uncertainty, transmission of the disease to the neonate, and neonatal separation [3,4]. An exploration of the psychological effects of the COVID-19 pandemic on pregnant women and the contributing factors is necessary to provide beneficial support to them and reduce adverse outcomes.

## Materials and methods

The present retrospective study was conducted in Bahrain Defense Force Hospital, a tertiary center in the Kingdom of Bahrain. The study design was approved by the Research Ethical Committee and Bahrain’s National COVID-19 Clinical Research Team. The participants included women who delivered term babies during the COVID-19 pandemic, whereas the control group comprised women who delivered term babies before the start of the COVID-19 pandemic. The period of study was between January 2021 and December 2021. For the study, researchers selected women who contracted COVID-19 within 15 days of delivery to be study participants. Infection was confirmed by nasopharyngeal samples using a polymerase chain reaction (PCR) test or SARS-CoV-2 GeneXpert. Participants’ existing mental health issues were excluded from the questionnaires and the computerized medical records. Throughout the study, the participants’ information remained confidential, and data were anonymized. The participants’ demographic characteristics were recorded.

The researchers used the Hospital Anxiety and Depression Scale (HADS) questionnaire to analyze levels of anxiety and depression, and it was distributed online to the study population 12 weeks postpartum. The questionnaire was explained verbally to each participant either in Arabic or in English, as well as in the language the participants preferred in order to understand the online questionnaire. The researchers scored participant responses separately for those pregnancies before and during the COVID-19 pandemic. The level of each symptom experienced was graded from 0 to 4, and then they were classified into normal (0-7), borderline abnormal (8-10), and abnormal (11-21). The researchers included the following contributing factors: social isolation, fear of disease transmission, fear of delivery, uncertainty of life, economic uncertainty, loss of family support, length of hospital stay, breastfeeding intervals, and neonatal separation.

Inclusion criteria

In the present study, we included multigravid women who contracted COVID-19 within 15 days of delivery, either before or during the pandemic. The researchers ensured that the chosen participants had neither personal nor familial history of mental health disorders and that they consented to participate in the study and understood the questionnaire well.

Exclusion criteria

The researchers excluded primigravid women who contracted COVID-19 during the pandemic and those who contracted a COVID-19 infection after their delivery. Furthermore, women who delivered preterm babies, had high-risk pregnancies, or had intrapartum complications were excluded, as were pregnant women with a history of mental disorders (including psychological stress, anxiety, and depression) before their pregnancy. Moreover, the researchers excluded those women for whom the following social factors were relevant: loss of family members, loss of family support, economic crises, and educational stress.

Statistical analysis

Continuous variables were expressed as mean ± standard deviation, and discrete variables were expressed as frequencies and percentages. Differences in scores before and during the pandemic were tested using a paired t-test. Associations between change in scores (during COVID and pre-COVID) and demographics and contributing factors were investigated using the analysis of variance (ANOVA) and t-test. Pearson’s correlation was used to investigate the relationship between change in scores and continuous variables, namely, length of stay, breastfeeding interval, and neonatal separation. The data were analyzed using SPSS Version 25.0 (IBM Corp., Armonk, NY). P-values of less than 0.05 were considered statistically significant.

## Results

A total of 111 women were included in the analysis, with an age range of 18 to 42 years (mean: 29 ± 6 years). Of them, 35.8% were aged <25 years, 47.2% were aged 25-35 years, and 17% were aged >35 years. In terms of BMI, 2.1% of them were classified as underweight, 16.7% as normal, 29.2% as overweight, 31.3 % as class I obese, 14.6% as class II obese, and 6.3% as class III obese (Table 1).

**Table 1:** Basic characteristics *P-value less than 0.05 †Calculated using analysis of variance or t-test as appropriate

Characteristic	N (%)	Change in anxiety score	P-value^†^	Change in depression score	P-value^†^
Age					
<25	38 (35.8)	15 ± 2	0.276	10 ± 3	0.192
25-35	50 (47.2)	15 ± 2		9 ± 3	
>35	18 (17)	16 ± 2		10 ± 3	
BMI					
Underweight (<18.5)	1 (2.1)	16 ± -	0.654	11 ± -	0.833
Normal (18.5–25)	8 (16.7)	14 ± 2		8 ± 2	
Overweight (26–30)	14 (29.2)	15 ± 2		9 ± 4	
Obese I (31–35)	15 (31.3)	15 ± 2		9 ± 2	
Obese II (36–40)	7 (14.6)	13 ± 2		8 ± 3	
Obese III (>40)	3 (6.3)	14 ± 5		9 ± 3	

To analyze levels of anxiety and depression, the HADS questionnaire was distributed to the selected population fitting our study criteria, and responses were used to analyze the score. During COVID-19, anxiety and depression in mothers increased significantly, with a mean difference of 14.97 (95% CI: 14.5 to 15.4) and 9.4 (95% CI: 8.8 to 9.9), respectively, with a p-value of <0.001 for both.

A Wilcoxon signed-rank test showed that the pandemic had a significant psychological effect in pregnancy during the COVID-19 pandemic (p<0.01) (Table 2). Looking at the results of the paired t-test of HADS, during the COVID-19 pandemic, both anxiety and depression had a significant p-value of <0.001 (Table 3).

**Table 2:** Summary of feedback to HADS before and after the start of the pandemic (N=111) ^a^Using Wilcoxon signed-rank test and based on negative ranks. HADS, Hospital Anxiety and Depression Scale

HADS	Median score		Test statistics^a^	
	Pre	Post	Z	P-value
Anxiety				
I feel tensed or “wound up”	0	2	-9.308	<0.01
I get a sort of frightened feeling like “butterflies” in the stomach	0	2	-9.276	<0.01
I get a sort of frightened feeling as if something awful is about to happen	0	2	-9.286	<0.01
Worrying thoughts go on in my mind	0	2	-9.318	<0.01
I can sit at ease and feel relaxed	0	2	-9.148	<0.01
I get sudden feelings of panic	0	2	-9.327	< 0.01
I feel restless as I have to be on the move	0	2	-9.217	<0.01
Depression				
I still enjoy the things I used to enjoy	1	2	-8.246	<0.01
I can laugh and see the funny side of things	0	2	-7.952	<0.01
I feel cheerful	0	2	-8.73	<0.01
I feel as if I have slowed down	0	2	-8.878	<0.01
I have lost interest in my appearance	0	2	-7.738	<0.01
I look forward with enjoyment to things	0	2	-8.565	<0.01
I can enjoy a good book or radio or TV program	1	2	-4.299	<0.01

**Table 3:** Paired t-test results of HADS score HADS, Hospital Anxiety and Depression Scale

HADS score (can range from 0 to 21)	Pre-COVID	During the pandemic	P-value
Anxiety	1 ± 1	16 ± 2	<0.001
Depression	4 ± 2	13 ± 2	<0.001

**Figure 1:**
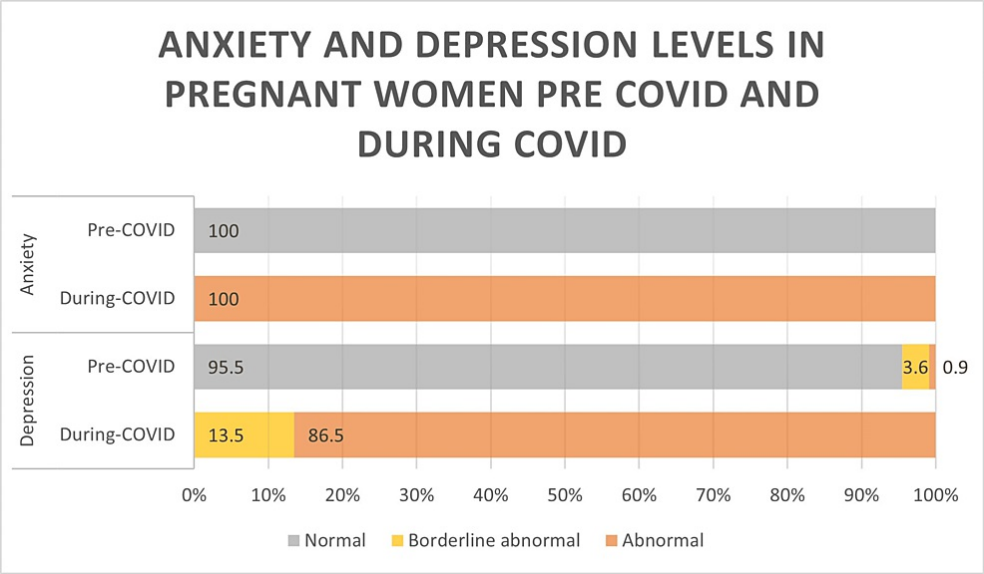
Illustration of the differences in anxiety and depression levels in pregnant women before and during COVID-19 (categorized as defined by the HADS scale). HADS, Hospital Anxiety and Depression Scale

Figure 1 shows the HADS questionnaire analysis and results in terms of levels of anxiety and depression in COVID-19 affected pregnancies and the pregnancies before the COVID-19 pandemic. The level of each symptom experienced was graded from 0 to 4, and then they were classified into normal (0-7), borderline abnormal (8-10), and abnormal (11-21).

Overall, 100% of participants who contracted COVID-19 during their pregnancy were classified as “abnormal” in the anxiety category. For the depression category, before COVID-19, 95.5% of them were classified as normal, 4% as borderline abnormal, and 1% as abnormal. During the COVID-19 pandemic, 86.5% were classified as “abnormal,” 15% were classified as borderline abnormal, and none of them had a normal score (Table 4).

**Table 4:** Differences in anxiety and depression levels in pregnant women before and during COVID (categorized as percentages)

	Anxiety		Depression	
	Pre-COVID	During-COVID	Pre-COVID	During-COVID
Normal	111 (100%)	0 (0.0%)	106 (95.5%)	0 (0.0%)
Borderline abnormal	0 (0.0%)	0 (0.0%)	4 (3.6%)	15 (13.5%)
Abnormal	0 (0.0%)	111 (100%)	1 (0.9%)	96 (86.5%)

Questionnaires included the following contributing factors: social isolation, fear of disease transmission, fear of delivery, uncertainty of life, economic uncertainty, and loss of family support (Table 5). High depression scores were significantly correlated to factors such as fear of disease transmission to the baby (p=0.027), fear of delivery (p=0.008), and loss of family support (p=0.001), whereas social isolation, isolation from family, uncertainty of life with disease, and economic crises did not significantly correlate to depression in our population. However, anxiety scores did not significantly correlate to isolation from social life, isolation from family, fear of delivery, fear of transmission of disease, uncertainty of life, loss of family support, and economic crisis in our study population. Contributing factors and anxiety scores yielded significant correlations with “fear of delivery” (r_s_=0.258), fear of transmission (r_s_=0.198), and uncertainty of disease life (r_s_=0.247). As for depression, it was significantly correlated to one factor: loss of family support (r_s_=-0.335).

**Table 5:** Association between change in anxiety/depression scores and demographics and contributing factors (mean ± SD). *P-value less than 0.05. †Calculated using analysis of variance or t-test as appropriate.

Contributing factors	N (%)	Change in anxiety score	P-value^†^	Change in depression score	P-value^†^
Isolation from family					
Yes	90 (81.1)	15 ± 2	0.827	9 ± 3	0.22
No	20 (18)	15 ± 2		10 ± 3	
Isolation from social life					
Yes	90 (81.1)	15 ± 2	0.26	9 ± 3	0.202
No	21 (18.9)	15 ± 2		10 ± 2	
Fear of transmission to baby					
Yes	76 (68.5)	15 ± 2	0.725	9 ± 3	0.027*
No	35 (31.5)	15 ± 2		10 ± 3	
Fear of delivery					
Yes	72 (64.9)	15 ± 3	0.059	9 ± 3	0.008*
No	39 (35.1)	14 ± 2		10 ± 3	
Uncertainty of disease life					
Yes	46 (43)	15 ± 3	0.072	9 ± 3	0.975
No	61 (57)	15 ± 2		9 ± 3	
Economic crisis					
Yes	16 (14.4)	14 ± 2	0.271	9 ± 2	0.916
No	95 (85.6)	15 ± 2		9 ± 3	
Loss of family support					
Yes	14 (12.6)	15 ± 2	0.575	7 ± 2	0.001*
No	97 (87.4)	15 ± 3		10 ± 3	

Associations of change in anxiety and depression scores with personal, social, and clinical contributing factors were investigated. The length of stay in the hospital, breastfeeding interval, and neonatal separation did not significantly affect both anxiety and depression in our study population (Table 6).

**Table 6:** Correlation of change in scores with length of stay, breastfeeding interval, and neonatal separation.

Contributing factors		Change in anxiety score		Change in depression score	
	Mean ± SD	Pearson’s correlation	P-value	Pearson’s correlation	P-value
Length of stay days	5.5 ± 3.1	0.048	0.619	0.068	0.481
Breastfeeding interval	8.4 ± 9.5	0.162	0.090	0.021	0.825
Neonatal separation	9.5 ± 8.7	0.137	0.152	0.014	0.888

## Discussion

Mental health is an integral part of people’s well-being. Psychological stress can occur from health, social, and economic factors, as well as lifestyle changes. Pregnant women may experience added. Researchers assessed the psychological impacts of COVID-19 infection during pregnancy and compared it to pregnancy before the pandemic.

The age of the study participants ranged from 18 to 42 years (mean = 29 ± 6 years). The researchers demonstrated that during the COVID-19 pandemic, anxiety and depression in pregnant mothers increased significantly, with a mean of 14.97 (95% CI: 14.5 to 15.4) and 9.4 (95% CI: 8.8 to 9.9), respectively (p<0.001). During 2021, Akgor et al. conducted a study of 297 pregnant women over the age of 18 years. Participants provided demographic and clinical details, resulting in a confidence interval of 95% from a Likert scale and a 14-item HADS questionnaire. The mean age of the participants was 27.64 years, and 82% had concerns about infecting their newborns with COVID-19. In the study, the researchers identified anxiety as a significant risk factor [5]. Furthermore, all participants who contracted COVID-19 during pregnancy were classified as “abnormal” in the anxiety category and 86.5% were classified as “abnormal” in the depression category, whereas before the pandemic, 0.9% of the studied population were abnormal, 3.6% were borderline abnormal, and 95.5% were normal in the depression category. Similarly, a cross-sectional survey in Qatar reported increased anxiety and depression during pregnancies and postpartum periods during the COVID-19 pandemic [6].

Furthermore, Saccone et al. conducted a cross-sectional study in Napoli, Italy, during 2019 to assess the impact of COVID-19 infections on 100 pregnant women using the the Italian version of the Impact of Event Scale - Revised (IES-R). The authors determined that the pandemic had a moderate psychological impact overall with a mean IES-R score of 36.9 ± 10.1%. Additionally, 53% of antenatal women noted a severe psychological impact of the COVID-19 pandemic [7]. In the present study, loss of family support was a significant contributing factor for increased depression scored during the COVID-19 pandemic; during the pandemic, quarantines and social distancing created barriers to some forms of support, which may have increased depression scores during this period.

Media focus on the pandemic, economic challenges, social isolation, quarantines, and decreased family interactions may have contributed to increased rates of psychological disturbances. Patabendige et al. during 2020 noted that pregnant women in Sri Lanka had an increase in perinatal anxiety and depression compared to their Chinese counterparts [8]. Furthermore, Zhang and Ma rated stress factors from the pandemic as moderate to severe among Chinese pregnant women between February and March 2020 using the IES-R and questionnaires assessing mental health, attitudes, and sociodemographic factors [9]. Furthermore, during 2020, Puertas-Gonzalez et al. conducted a cross-sectional study with 200 pregnant participants to explore the psychological effects of the COVID-19 pandemic on antenatal women. The authors compared two groups (pregnancies before and during the pandemic, respectively) of 100 antenatal women and found that the group that gave birth during the pandemic had higher scores in the depression dimension of the Symptom Check List-90 survey, increased phobic anxiety, and increased perceived stress. Insomnia was found to be an important explanatory variable in the Perceived Stress Scale [10].

In the present study, fear of delivery and fear of disease transmission were significant contributing factors to high anxiety scores, regardless of adequate intrapartum care and support. The researchers reviewed five databases, including 13 articles written by Brooks et al., highlighting that uncertainty, disrupted routines due to infection fears, financial concerns, and health care issues must be addressed. Updated information, support, guidance, and advice should be provided to pregnant women regarding the different risks and benefits of treatment [3]. However, the results of the present study indicated no statistical significance related to social isolation, uncertainty of life, and economic crises.

In 2020, Shahid et al. conducted a descriptive cross-sectional study including 552 pregnant women, irrespective of parity and gestational age, to analyze the psychological impacts of COVID-19 infection during pregnancy. The authors used a Kessler-10 scale and the Edinburgh Postnatal Depression Scale (EPDS) to assess anxiety and depression. The authors reported that issues of fear of vulnerability to COVID-19 infections and disease transmission to newborns led to an increase in psychological stress, anxiety, insomnia, and depression in the study group [11]. This result aligns with that of the present study. Additionally, Zanardo et al. conducted a non-concurrent case-control study comparing pregnant women who delivered babies during the COVID-19 pandemic, from March 8 to May 3, 2020, to a matched group who delivered during this same time period in 2019. The authors found that the pandemic group showed markedly higher EPDS scores and more anhedonia and depression compared to the control group [12]. Furthermore, during 2020, Parra-Saavedra et al. conducted a cross-sectional web survey related to demographic, knowledge, and attitude data, as well as the psychological symptoms of 946 pregnant women from seven cities in Columbia and found that 50.4% had anxiety, 49.1% had sleep disturbances, and 25% had depression [13].

A cross-sectional study in conducted 2021 by Jelly et al. including 333 pregnant women in Uttarakhand, India, found that the COVID-19 pandemic did not cause negative psychological effects [14]. During 2021, Lopez-Morales et al. conducted a longitudinal study including 102 pregnant women and 102 non-pregnant women to analyze the psychological effects of the COVID-19 pandemic on both groups of women. In the study, the group of pregnant women showed an increase in anxiety, depression, and overall negative effects compared to the non-pregnant group [15]. Similarly, Fan et al. conducted a meta-analysis of 19 articles from four databases till September 27, 2020, to study the mental health status of pregnant women. The authors concluded that there was a 42% prevalence of anxiety and 25% for depression. Younger antenatal women, that is, those aged 18-25 years, were more anxious. Social support and physical activity may decrease the likelihood of depression and anxiety [16]; hence, appropriate measures should be taken to ensure that pregnant women have a safe pregnancy and postpartum period. Current telecommunication services for pregnant women provide adequate support to reduce fear of disease transmission and adverse outcomes. In the present study, HADS anxiety and depression scores illustrated that pregnant women during the COVID-19 pandemic experienced significantly higher negative psychological effects than those women pregnant before the pandemic (Table 2).

Milne et al. conducted a study of 70 pregnant women who completed a questionnaire asking about their mood and relationships during COVID-19-related isolation periods. Almost (95.7%) all of the women did not report relationship deterioration, although 44% experienced lowered mood due to the lack of contact with family and friends. Additionally, 14% experienced an increase in anxiety as a result of financial burden due to the inability to work. On a positive note, 34% felt that the isolation was relaxing [17]. In a study by Moyer et al. during 2020, a total of 2,740 pregnant women were surveyed online to assess the impact of the COVID-19 pandemic on antenatal women’s anxiety and the factors that caused it. The authors found that the number of individuals planning a hospital delivery dropped from 2,641 to 2,400 during the COVID-19 pandemic. Additionally, stress regarding food shortages was experienced by 59.2%, stress from job loss was experienced by 63.7%, and stress from loss of childcare was experienced by 56.3%. Moreover, 37.5% reported increased stress due to conflicts in the household, and 93% had a fear of contracting an infection [18].

Ostacoli et al. conducted a cross-sectional study during 2020 on the factors causing psychological distress in the postpartum period during the COVID-19 pandemic. In an online survey, 163 women shared their parturition experiences and their perception of the effects of the pandemic. The authors found that postpartum depression and posttraumatic stress symptoms were higher in women who delivered during the COVID-19 pandemic [19]. Similarly, in 2021, Pacheco et al. noticed dwindling mental health in women with unpleasant breastfeeding experiences due to a lack of family and professional support [20]. Spinola et al. in their study during 2020 concluded that newborn admission to the neonatal intensive care unit contributed to higher levels of postpartum depression and anxiety for mothers [21]. In the present study, the researchers found that the mean length of stay in the hospital was 5.5 ± 3.1 days, the breastfeeding interval was 8.4 ± 9.5 days, and the length of neonatal separation was 9.5 ± 8.7 days. These factors were not statistically significant. The researchers emphasize that providing psychological intervention to women falling pregnant and giving birth during the COVID-19 pandemic may prevent further adverse outcomes.

 Study strength

To match the variables of the samples, the researchers selected women who contracted COVID-19 within 15 days of delivery and compared the psychological stress of the women who were infected and delivered during the COVID-19 pandemic and those who delivered before the COVID-19 pandemic. The researchers excluded women who delivered at less than 37 weeks of gestational age, those who had high-risk pregnancies, and those who had intrapartum complications. The questionnaires were distributed after the postpartum period of 12 weeks. During this period, all the participants completed their respective quarantine periods and resolved their stress and fears of COVID-19 during the pregnancy. The questionnaires were explained well to the participants verbally as well as online, and the feedback was collected.

Limitations

The HADS responses were provided to the researchers via self-grading of symptoms by the participants. As such, errors in recalling such symptoms and their severity by the study population are possible. Additionally, researchers did not include women infected with COVID in all trimesters. Future studies could include this factor to study variation in psychological effects according to gestational age. Furthermore, we selected only women who contracted COVID-19 during their third trimester.

## Conclusions

The researchers’ assessment of the mental health of pregnant women during the COVID-19 pandemic showed that the pandemic had a negative psychological effect on these women, increasing both anxiety and depression. Contributing factors included loss of family support, fear of delivery, and fear of disease transmission. Other factors, including social isolation, uncertainty of life, economic hardship, length of hospital stay, neonatal separation, and breastfeeding intervals, did not demonstrate significant correlations with women’s psychological well-being. A comprehensive analysis of the mental health issues suffered by women pregnant during the COVID-19 pandemic and the contributing factors is necessary to provide this population with adequate support and reduce adverse outcomes.
